# Key Factors Regulating the Interdomain Dynamics May Contribute to the Assembly of ASC

**DOI:** 10.3390/biology12060796

**Published:** 2023-05-31

**Authors:** Tongtong Li, Laura I. Gil Pineda, Amy O. Stevens, Yi He

**Affiliations:** 1 Department of Chemistry & Chemical Biology, The University of New Mexico, Albuquerque, NM 87131, USA; litt@unm.edu (T.L.); lgilpineda@vt.edu (L.I.G.P.); ao630@unm.edu (A.O.S.); 2 Department of Biochemistry, Virginia Tech, 340 West Campus Dr., Blacksburg, VA 24061, USA

**Keywords:** apoptosis-associated speck-like protein containing a CARD (ASC), inflammasome complex, interdomain dynamics, interdomain rotation, flexible linker, type I interaction

## Abstract

**Simple Summary:**

The innate immune system in our bodies responds to pathogenic infections and cellular damage by inducing pyroptosis through the assembly of inflammasome complexes. The apoptosis-associated speck-like protein containing a CARD (ASC) serves as an adapter, recognizing pattern recognition receptors (PRRs) and procaspase-1 based on the homotypic interactions of the pyrin domains (PYD) and the caspase recruitment domains (CARD) within the inflammasome complexes. The structural diversity of ASC and the role of the semi-flexible linker in structural transitions are critical in understanding the biological functions of ASC. This study employs molecular dynamics simulations to explore the structural dynamics of ASC and to analyze the potential relationship between interdomain dynamics and the biological roles of ASC as an adapter. The findings suggest that ASC dynamics partially originate from the movement of the linker and that the type I interaction surface on PYD is generally exposed and inaccessible to the CARD domain. These insights are consistent with experimental results and shed light on the function-related dynamic behaviors of ASC.

**Abstract:**

The canonical ASC domains, PYD and CARD, are interconnected by a lengthy, semi-flexible linker. The molecular basis and purpose of ASC’s highly dynamic feature remain elusive. In this study, all-atom molecular dynamics simulations were utilized to examine the role of the linker and the interdomain dynamics of the ASC monomer. As revealed in the principal component analysis (PCA), the flexible linker enables interdomain dynamics and rotation. The stumbling between domains is partially attributed to the helical portion of N-terminal residues in the linker. Additionally, the linker exhibits a certain structural preference due to the turn-type structural inclination of the N-terminal and the presence of several prolines on the linker. Such structural preferences lead to the unavailability of regions for PYD type I interactions to CARDs, as evidenced by the CARD spatial restraint analysis. In conclusion, the semi-flexible linker introduces functionally relevant interdomain dynamics, potentially enhancing PYD self-assembly and the subsequent assembly of the inflammasome complex.

## 1. Introduction

The immune system can rapidly recognize and respond to damage- or pathogen-associated molecular patterns (DAMPs and PAMPs) in order to maintain homeostasis and promote health. These DAMPs and PAMPs can be detected by host pattern recognition receptors (PRRs), such as NOD-like receptors (NLRs) and absent in melanoma 2 (AIM2), via inflammatory pathways [1,2,3]. PRRs then activate the immune response by triggering procaspase-1 and forming the inflammasome complex through an adapter protein named apoptosis-associated speck-like protein containing a CARD (ASC) [1,4]. ASC recruits PRRs and procaspase-1 through homotypic interactions of its pyrin domain (PYD) and caspase recruitment domain (CARD) domain with the PYD-containing PRRs and the CARD of procaspase-1 [1,4,5,6]. Inflammasomes can vary depending on the types of PRR; four inflammasome complexes have been extensively discussed in a previously published review article [7]. In addition to acting as adapters in the inflammasome platform, ASC can oligomerize into what is known as the ASC speck to amplify immune response signaling by creating multiple activation sites [8]. The dysfunction of ASC has been associated with different types of cancer [9,10,11,12], diabetes [13], gout [14], and Alzheimer’s disease [15], among others [16,17,18,19,20].

**Figure 1:**
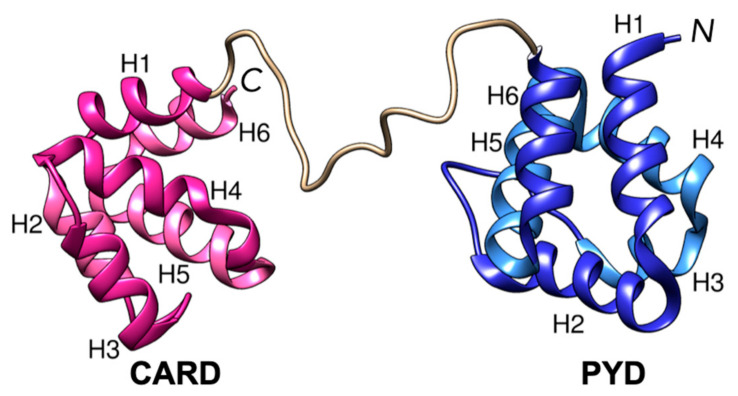
Apoptosis-associated speck-like protein containing a CARD (ASC) full-length monomer, containing the pyrin domains (PYD) and the caspase recruitment domains (CARD). Each domain consists of a 6-helix bundle, and PYD and CARD are connected by a 23-residue linker.

Human ASC is a protein comprised of 195 amino acids (Figure 1). The solution NMR structure has shown that the PYD and CARD domains are structurally independent six-helix bundle motifs connected by a 23-residue semi-flexible linker. The dynamics between these domains are essential for linking the functions of PYD and CARD so they can work collaboratively. Previous work by de Alba used NMR relaxation techniques [21] to indicate that the dynamic behavior of the PYD and CARD of ASC lies between two extreme models (i.e., the PYD and CARD neither tumble as a single rigid body nor are the domains dynamically independent), showing interdomain flexibility while simultaneously sensing the drag of the other [21]. With this, the length of the linker could be an essential determinant in the interdomain dynamics and the function of death domain proteins [21]. Furthermore, the flexible linker positioned between the PYD and CARD of ASC is also vital for the globular ASC speck formation. Bryan et al. demonstrated that ASC-b, an alternative splicing variant of ASC that lacks the flexible linker, could still form specks when co-expressed with NLRP3. However, these specks were not as compact, and the IL-1b maturation was less efficient. When co-expressed with ASC-a (full length), ASC-b interfered with the formation of normally condensed specks and instead initiated the formation of larger, irregular-shaped specks [22]. In a separate study, the alternative splice variant of ASC failed to form the ASC speck and instead formed filaments [23]. Within the assembly of ASC filaments [24,25,26], a model was proposed based on the cryo-EM structure of ASC PYD filaments [24], combined with the solution NMR structure of the full-length ASC [21], wherein ASC PYDs self-assemble into filaments, and ASC CARDs protrude outward [24]. Another model suggests that both PYD and CARD in ASC contribute to the self-assembly of ASC based on the filament size measured in the TEM results [25]. It is widely accepted that homo-/hetero- oligomerization of PYDs is critical to the formation of inflammasome complexes. Interestingly, a sensor protein named NLRP9 was discovered to exist as a monomer in vitro [27,28,29]. The isolated state of NLRP9, containing an N-terminal PYD and a NACHT domain, is presumed to attribute to the unique structural features of NLRP9 PYD. One potential factor could be the charge inversion of residues at the interacting interface of NLRP9 PYD [27]. Another factor may be residues at the N-terminus of NLRP9 PYD that can interact with helix 1, thus disrupting the type I interface composed of helices 1 and 4, and subsequently inhibiting PYD self-assembly [28]. In light of this N-terminus autoinhibition hypothesis, regions in ASC domains that interact with the linker may be partially shielded from contact with other proteins, while other regions remain free to participate in self-assembly or protein–protein interactions. A comprehensive discussion on activation, the formation of inflammasome complexes, and regulatory mechanisms can be found in a recent review [30].

Atomic resolution information on structure and dynamics is pivotal for understanding the fundamental physicochemical properties of the multi-talented ASC. Previous NMR experimental studies have shown that the ASC linker governs its interdomain dynamics by partially restricting the accessible conformational space of each domain relative to the other [21]. In this paper, we study the interdomain dynamics of ASC at atomic resolution in silico by performing extensive simulations of the ASC monomer and the isolated 23-residue linker fragment of ASC, with explicit solvent, using the CHARMM 36 force field. Our principal component analysis (PCA) results suggest that the interdomain dynamics of ASC monomer are encoded by the linker. The motion patterns in the ASC monomer are similar to those in the isolated linker, including the interdomain rotation and the change of relative positions. Notably, CARD does not have access to the interfaces responsible for PYD type I interactions, typical interaction regions in the ASC assembly. This suggests that PYD assembly appears to benefit from the dominant interdomain dynamics via the semi-flexible linker. Furthermore, we delved into the possible factors affecting the interdomain dynamics. We posit that the structural preference at the N-terminus of the linker and the presence of several prolines may play a significant role in ASC interdomain dynamics.

## 2. Materials and Methods

### 2.1. All-Atom Simulation Details

Two systems were simulated: the ASC full-length monomer (195 residues), as shown in Figure 1, and ASC linker (residues 90–112, 23 residues). Both structures were obtained from the solution NMR structure of ASC (PDB ID: 2KN6) [21]. All simulations were performed using the CHARMM 36 force field with explicit solvents and the Groningen Machine for Chemical Simulations (GROMACS) package [31,32,33] version 2018.3. Each protein was placed in a cubic box of TIP3P water, along with counter ions (K^+^ or Cl^−^) to neutralize the whole system at 300 K. Long-range electrostatics were calculated using the particle-mesh Ewald (PME) algorithm [34,35]. Periodic boundary conditions were applied in all directions. Each system of protein, water, and counterions was prepared using CHARMM-GUI [36,37], which generates a series of GROMACS inputs for subsequent MD simulations. To generate equilibrated starting structures for the MD simulations, after placing each protein in a water box with counterions, steepest-descent minimization was carried out, followed by a 1 ns MD simulation with a time step of 1 fs, to heat the whole system from 1 K to the desired temperature. All bonds with hydrogen atoms are converted to constraints with the algorithm LINear Constraint Solver (LINCS) [38], using the default parameters of the GROMACS package. The equilibrated structures obtained from the aforementioned steps were used for subsequent production runs. A Nose–Hoover temperature thermostat [36,37] was used to maintain the temperature. The time step was set at 2 fs, and snapshots were taken every 100 ps. For the ASC monomer, a cubic water box size of 110 Å was employed and run for a total of 22 µs including five 3.2 μs long MD trajectories and six 1 μs long MD trajectories. As for the ASC linker, a similar setup was used but with a different water box (a cubic water box size of 80 Å) for a total of 27 µs including nine 3.0 μs long MD trajectories.

### 2.2. Cluster Analysis and Time-Resolved Force Distribution Analysis (TRFDA)

The hierarchical cluster analysis was performed using Ward’s minimum variance method [39]. It is based on the PCA [40] results, whereby the first two principal components were used in cluster analysis to determine 5 representative clusters from all 22 µs of ASC monomer simulations. TRFDA [41,42] can be used to probe the forces on an atomic level when macromolecules undergo conformational transitions. Based on the pairwise forces between atoms, we can determine the forces on a specific residue of interest due to its interactions with other residues, referred to as the punctual stress. In this work, the punctual stresses were averaged every 20 frames for six 1 µs trajectories of the ASC monomer. TRFDA helped to trace the dynamics of internal forces on each residue during ASC monomer dynamics. Specifically, TRFDA was employed to capture the interactions between PYD-CARD and between the linker and either PYD or CARD. This will help us to identify the key residues enduring high punctual stresses during the conformational dynamics.

### 2.3. Other Structural Analyses

Distance and angle analyses were performed using the GROMACS package [43]. The center of mass (COM) distance between domains PYD and CARD was calculated by measuring the distance between COM of two regions, residues 1–89 (PYD) and residues 113–195 (CARD). Root mean square fluctuations (RMSF) and secondary structural analysis were computed using MDTraj [44]. Principal component analysis (PCA) and correlation analyses, conducted using bio3D [40], aided in visualizing the principal components and exploring the correlation of motions between any pair of residues, shedding light on protein dynamics. To examine the spatial distribution of CARD domain around the PYD domain, we aligned the PYD domain and colored the CARD domain in grey, with a darker grey representing a higher population of that particular conformation.

## 3. Results

### 3.1. Highly Dynamic ASC Monomer and the Semi-Flexible Linker

#### 3.1.1. ASC Monomer Is Highly Dynamic

**Figure 2:**
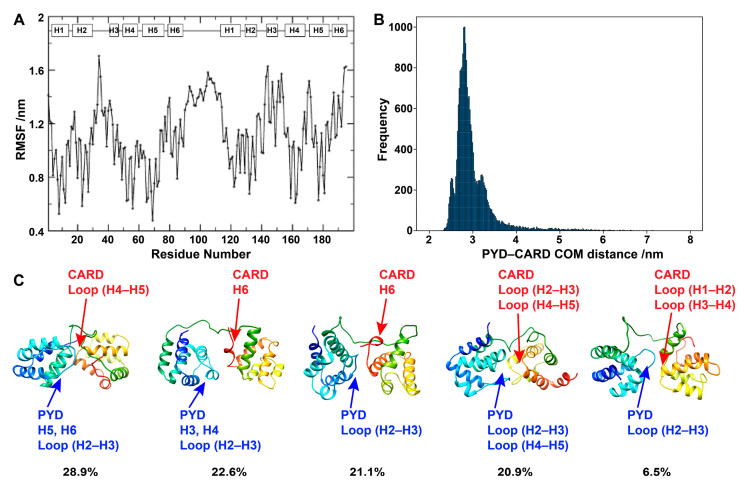
(**A**) Root mean square fluctuations (RMSF) and (**B**) distribution of the center of mass (COM) distance between domains PYD and CARD for ASC monomer simulations, for the whole 22 µs trajectory. (**C**) Five representative ASC monomer structures obtained from cluster analysis roughly display the contact interfaces between PYD and CARD, with some physically adjacent regions of domains PYD and CARD labeled on the side.

As observed from the ASC monomer simulations, there is no stable packing structure between PYD and CARD. As shown in Figure 2A, several regions in the ASC monomer are highly flexible, such as the loop region between H2 and H3 of the PYD domain (residues 30–40), the linker (residues 90–112), and a region in the CARD encompassing H3 plus a portion of H4 (residues 143–157, where helix 4 in the CARD spans residues 155–168). The distance between the center of mass (COM) of PYD and CARD varies between 2–8 nm, primarily settling at a range of 2.5–3.5 nm (Figure 2B). The monomer occasionally adopts extended structures with COM distances exceeding 6 nm, potentially acting as transition structures. These structures may facilitate the interdomain dynamics and tuning of the relative positions of the domains. It should be noted that the same COM distance does not always correspond to the same structure, as substantiated by subsequent PCA-based cluster analysis. Figure 2C presents five representative structures derived from the cluster analysis. These illustrate the considerable flexibility of the ASC monomer in assuming different conformations, especially given that the contact interfaces between PYD and CARD comprise varying regions from each domain. These structures suggest that the linker can partially enclose the PYD and CARD domains. Notably, the interfaces for the type I interactions (the regions from blue to cyan in Figure 2C) are seldom obscured by the linker. In Figure S1, the broad distribution of the torsional angle, defined by the Cα atoms of residues 81–69–163–177 (as shown in Figure S1D), as well as the bending angle, defined by the Cα atoms of residues 49–69–163 (as shown in Figure S1E), indicate a wide spectrum of accessible interdomain motion and rotation.

**Figure 3:**
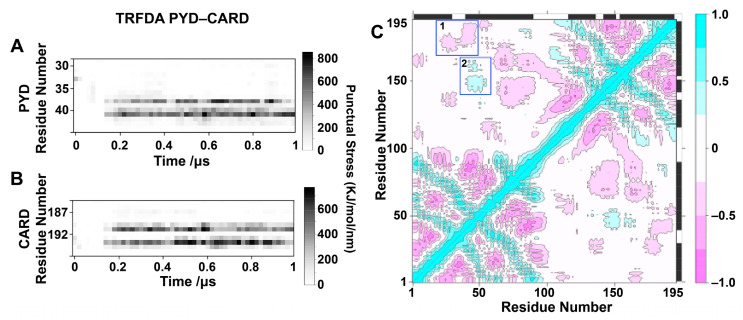
The time-resolved force distribution analysis (TRFDA) between domains PYD and CARD from the six 1 µs trajectories, and the regions with high punctual stress are located at PYD residues 38–41 ((**A**), covering the region of the loop between H2 and H3) and CARD residues 190–194 ((**B**), covering the region of the loop between H5 and H6). (**C**) Correlation analysis corresponding to all 22 µs of ASC monomer simulations. Light blue and pink in the scale bar indicate positive and negative correlations, respectively. The positive and negative correlation denotes two residues prefer to move in the same and opposite directions, respectively. The blue boxes highlighted regions 1 and 2, displaying an interesting positive/negative correlation.

To delve deeper into the interdomain dynamics, a thorough analysis of the ASC monomer was conducted, employing time-resolved force distribution analysis (TRFDA) and correlation plots. TRFDA is a useful tool for detecting residues under significant punctual stress, which are integral to maintaining the protein’s structural integrity and contribute to the ASC interdomain dynamics. Here, TRFDA was applied to evaluate the interaction between PYD and CARD using six 1 µs trajectories. The punctual stress was averaged over the six trajectories. Figure 3A,B depict residues under high point stress, such as residues 38–41 in the loop region connecting H2 and H3 of PYD and residues 190–194 in the H6 of CARD. A comprehensive TRFDA between PYD and CARD, in addition to the TRFDA between the linker and either PYD or CARD, is available in the Supplementary Materials (Figures S2 and S3). The correlation plot (Figure 3C) draws attention to two regions of interest. The first is a region of negative correlation (1-pink) between the loop region connecting H2 and H3 of PYD and H5 and H6 of CARD. This implies that these PYD and CARD regions tend to move in opposite directions. This is important because it corresponds to the residues under high punctual stress shown in Figure 3A,B. Thus, high punctual stress, possibly related to transient contacts, may be a contributing factor to the monomer dynamics. The second is a region of positive correlation (2-light blue) between H3 and H4 of PYD and H3 and H4 of CARD. The positive correlation suggests that these PYD and CARD regions tend to move in the same direction. This is a pertinent finding because after generating a network using the correlation data (Figure S4), it was noted that this CARD region is the only one where residues are correlated to PYD (blue) or not correlated with any of the domains (residues 141-green and 143-yellow). Moreover, PYD is predominantly correlated to the first nine residues of the linker. An interesting discovery was the identification of two distinct residues in the linker (residues 99 and 100) that do not correlate with either domain. This was later confirmed by subsequent PCA results. Furthermore, the relative average solvent-accessible surface area (SASA) for all helices (Table S1) within PYD and CARD was calculated to qualitatively estimate the interplay between the helices with the other domain or the linker. As shown in Figure S5, the relative SASA results indicate that the SASA of H3 in PYD and H5 and H6 in CARD was significantly lower than the rest of the helices in the ASC simulation. This is consistent with the TRFDA and correlation results, indicating that the loop region connecting H2 and H3 in PYD and H6 in CARD experienced high point stress, which hints at potential transient interactions. These findings suggest a higher probability for these regions to be buried.

The ASC monomer is highly dynamic, with its PYD and CARD domains arranged differently in terms of spatial positions and orientations. The loop regions in the two domains and the linker demonstrate significantly more fluctuation compared to other regions. One domain interacts with varying surfaces of the other domain across different clusters. TRFDA results suggest some weak interactions between PYD and CARD, with the motion of these two domains showing correlation in certain segments. In order to further understand the dynamics of the ASC monomer, we have isolated the linker from the simulation for a detailed analysis, which we will present in the following section.

#### 3.1.2. Linker Has Structural Preferences but Is Still Highly Dynamic

**Figure 4:**
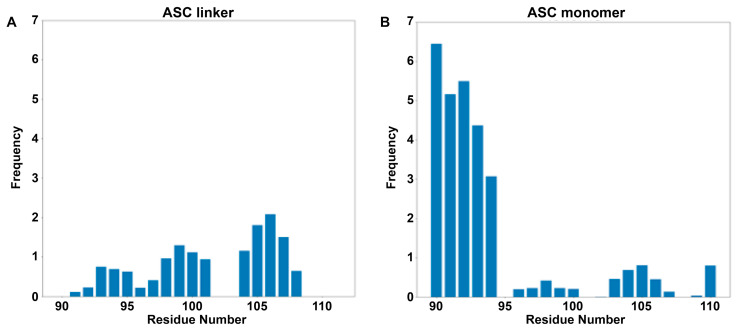
The secondary structural preference of the linker in the ASC linker (**A**) and monomer (**B**). The y-axis is the frequency (%) of each residue adopting a helical structure for each system.

The linker, as observed in the TRFDA results (Figure S3B,D) and correlation analyses (Figure 3C), is closely related to the dynamics of the ASC monomer. The first step in understanding the role of the linker (residues 90–112) in ASC dynamics was to determine if the linker possessed structural preferences that could influence the orientation of the domains or impose movement restrictions. A prior study suggested that the linker exhibited some local structure because of its short-range NOEs [21]. The same study determined that residues 90–94 displayed some positive ^13^C_α_ secondary chemical shifts, implying the possible adoption of turn-type conformations. In contrast, residues 95–112 had primarily negative ^13^C_α_ chemical shifts, indicating the formation of sparsely populated extended structures. We utilized the DSSP program [45,46] to ascertain the secondary structure preference of each residue in the linker region for both the ASC free linker and monomer simulations. Our objective was to determine if the presence of the domains influenced the linker’s structure arrangement. As depicted in Figure 4A,B, there are differences between the DSSP outputs of each system. There seems to be minimal structural preference in the ASC linker segment, while the linker in the ASC monomer exhibits a higher helical preference in residues 90–94, compared to the remaining residues of the linker. However, a subsequent analysis of the linker revealed the ten-residue stabilization core (continuous residues with the lowest fluctuation) to be residues 94–103 (Figure S6) rather than residues 90–94, which exhibited higher structural preference. This indicates that residues 90–94 remain highly dynamic, despite their structural preference. A five-residue core lies between residues 97–101, including residues 99 and 100, which did not correlate to any domains in the ASC monomer analysis. Observing the sequence of the linker, there are several prolines present, such as Pro97, Pro103, Pro104, and Pro110. These residues limit the degree of freedom of dihedral angles of adjacent residues, leading to increased rigidity in the linker. Therefore, both the turn-type preference and the existence of prolines result in some degree of restricted flexibility in the linker.

Though the linker exhibits some structural preference, its probability is relatively low. Consequently, a more in-depth analysis of the linker’s flexibility is required. As demonstrated in Figure S7A, a few weak contacts in the contact map demonstrated that the linker is likely more flexible rather than forming a stable structure. The flexibility of the linker is also confirmed by the negative correlation between N/C-terminus in the correlation map (Figure S7B). We subsequently characterized the end-to-end distances of the linker (the distance between residues 90 and 112) and the opening/closing degree of the linker (the dihedral angle defined by the Cα atoms of residues 90–99–100–112) for ASC linker and ASC monomer. As shown in Figure S8, the wide distance and angle distributions further suggest that the linker is dynamic.

The linker is highly dynamic, but we do observe the low-populated structures with a turn-type structure transiently formed at its N-terminus, especially in the monomer. In combination with the presence of prolines, the linker is not fully flexible but displays a structural preference to some extent.

### 3.2. Interdomain Dynamics and Its Origin

**Figure 5:**
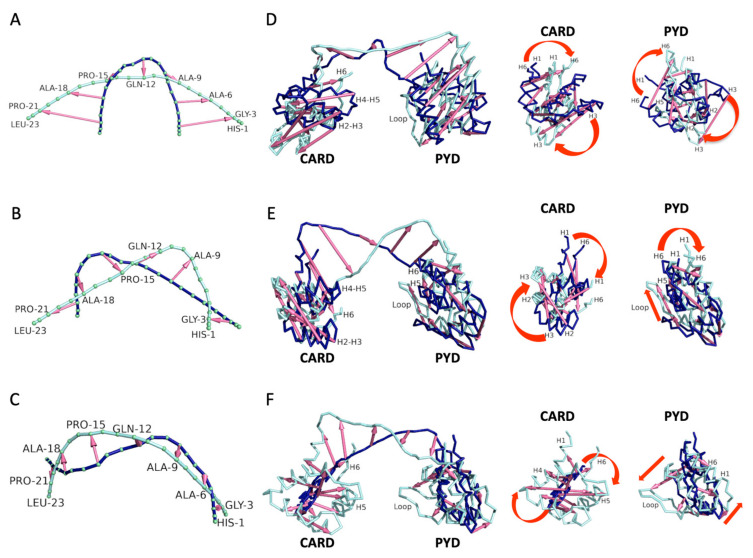
Principal component analysis (PCA). Dark blue is the initial structure and light blue is the final structure. (**A**–**C**) First three principal components for ASC linker simulations. (**D**–**F**) First three principal components for ASC monomer simulations. A close-up is presented for each domain to illustrate the effect that the linker movement has on the domains. “Loop” refers to the loop between H2 and H3 of PYD.

To study the interdomain dynamics, it is essential to conduct principal component analysis (PCA). In Figure 5, we have the first three PCA results for two systems: ASC linker (Figure 5A–C) and ASC monomer (Figure 5D–F). To analyze the movements, the dark blue structure is employed as the initial structure while the light blue structure represents the final state. However, it is crucial to recognize that the systems are actually switching between these two states. We have identified three key movements within the linker, which can be analogized to chemical bond vibrations: (i) bending in a scissoring fashion, (ii) a pseudo-asymmetrical stretching, and (iii) out-of-plane bending in a twisting fashion. From a residue-specific perspective, residues 99 and 100 are central to these movements, potentially explaining their lack of correlation with either domain. Moreover, we see two opposing movements lead by the first nine residues (~90–98 in monomer = 1–9 in linker) and the final seven residues of the linker (106–112 in monomer = 17–23 in linker). These observations clarify their negative correlation.

Moving on to the ASC monomer, the linker region has a very similar movement to the one described above, albeit with some distortion in shape. It is important to analyze how the movement of the linker is coupled with changes in rotation and orientation of the PYD and CARD domains. As shown in Figure 5D, when the linker “opens”, it prompts the PYD to rotate, causing its H1 and H6 to recede from CARD, while H2 and H3 advance towards it. Simultaneously, CARD itself rotates, resulting in its H1 and H6 drawing closer to PYD, while H3 recedes. These rotations induce a change in orientation of the domains such that the loop between H2 and H3 (loop H2–H3) of PYD and H6 of CARD are now facing each other, explaining their (loop H2–H3 and H6) negative correlation. Of course, this could be described in the reverse direction (when the linker “closes”), leading to an orientation where PYD’s H6 and H5 face the loops of H2–H3 and H4–H5 of CARD. This is more evident in the subsequent movement (Figure 5E), where the interface formed by H6 and H5 of PYD is highlighted in the initial structure. The overall orientation change in this second movement is similar to the first. However, the third movement involves a twisting movement, driving the domains to opposite ends of the plane. To illustrate this, CARD appears to be distorted as it is first thrown back (small) and then moved forward (enlarged). Its initial orientation is not entirely clear, but at the end of the movement, we find CARD’s H6 and H5 oriented towards PYD, with CARD’s H1 and H4 on the opposing side. As for PYD, we once again see loop H2–H3 facing CARD.

While observing these varied movements, it is important to emphasize that the interfaces formed by H1–H4 and H2–H3 of PYD (Type I interaction) are consistently maintained at a distance from CARD. Previous NMR work [47] proposed that these helices serve as sites for the initial PYD-PYD interaction in the assembly of ASC PYD filaments. The same work found that these helices are also the binding site for NMRP3 PYD [47]. Thus, our molecular dynamics simulation analysis indicates that within the extensive dynamic behavior of ASC, there is a conformational bias for the PYD to freely interact through the Type 1 interface. This conclusion aligns with a previous hypothesis proposed from NMR relaxation studies of full-length ASC [21].

**Figure 6:**
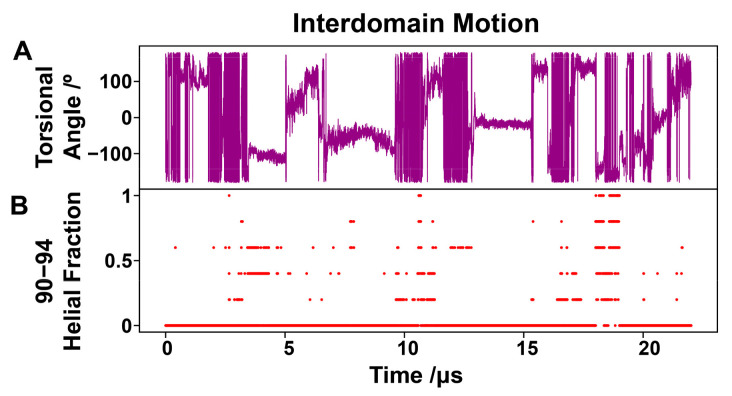
ASC interdomain dynamics and the helical preference of short segment at the N-terminus of the linker. The torsional angle determined by residues 81–69–163–177 (**A**) and the helical probability of residues 90–94 at the N-terminus of the linker (**B**) vary over time.

The interdomain movement was further evaluated by analyzing the torsional angle illustrated in Figure S1D. The Cα atoms of four residues (residues 81 and 69 in PYD domain and residues 163 and 177 in CARD domain) were used to define this torsional angle. This allowed us to characterize the interdomain rotation. While the torsional angle remained consistent at times, it also exhibited periods of swift transition between −180°–180°, as observed in Figure 6A. This is consistent with the observation of the interdomain relative rotation in PCA. Aiming to ascertain the origin of the relative instability of the ASC monomer, we found that the interdomain rotation may be associated with the transient helical structure in the N-terminal region (residues 90–94) of the linker. The helical propensity of residues 90–94 was derived based on the DSSP results, and the propensities 0 and 1 represent none and all residues are in a helical structure, respectively. When tracing the time evolution of the torsional angle 81–69–163–177 and the helical propensity of residues 90–94 (Figure 6A,B) simultaneously, it is intriguing to note that as the helical propensity changes, the torsional angle fluctuates between −180° and 180° frequently. The interdomain relative rotation, as displayed in the variation of the torsional angle, seems to result from the dynamic formation/destruction of the short turn-type/helix conformations in residues 90–94 (Figure 6B). However, the structural preference is relatively weak, so there may be a weak correlation between the transient helix in the linker and the interdomain rotation. It is likely other factors will also affect the interdomain stumbling of the ASC monomer.

PCA results on the isolated linker and ASC monomer reveal that the interdomain dynamics of ASC originate from the linker. Moreover, interdomain dynamics are accompanied by interdomain rotation. We characterize the interdomain rotation using a torsional angle, as shown in Figure S1D, and propose that the variation in this torsional angle is partly attributable to the turn-type status at N-terminal residues of the linker.

### 3.3. Interdomain Dynamics and Its Potential Role in ASC Self-Assembly

De Alba previously proposed that the ASC interdomain topological organization aids binding by avoiding steric interference between the two interacting regions of both domains and by favoring a specific protein binding orientation [21]. Although our PCA result supports this, we sought to further validate it by examining the space around PYD that CARD can access.

**Figure 7:**
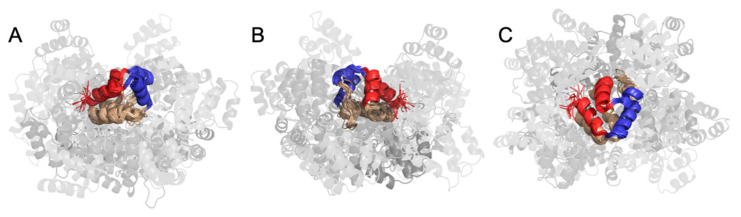
The results for the spatial distribution of CARD are shown here. PYD has been aligned: in red is the region covered by H1 and H4 and in blue is the region covered by H2 and H3, which make up the Type I interaction surfaces. CARD and linker surround PYD in every conformation found. The darker the conformation implies there is a higher probability of accessing that region or space. (**A**) One side view. (**B**) Side view from the opposite end. Here, we see the most probable conformation adopted by CARD, which is below the beige region of PYD (H5 and H6). (**C**) The top view that shows how CARD can widely cover the space below PYD and has some range (although with low probability) around PYD as well.

We utilized the acquired frames to cluster the structures and calculate the probability of each spatial arrangement of CARD (Figure 7). This showed that the Type I interaction region in PYD was almost inaccessible to CARD, and those structures where CARD was in close proximity to the region had the lowest probabilities. This aligns with the distribution of the bending angle defined by the Cα atoms of residues 49–69–163 (Figure S1C), ranging from 30° to 160°. The structure with a bending angle in the range of 0°–30° is lowly populated, which represents some structures where CARD occurs in the surroundings of the PYD H1–H4.

The spatial distribution of CARD around PYD implies that the ASC dynamics contribute to its function as an adapter in the inflammasome. In general, the Type I interaction on the surface of PYD, which is responsible for ASC self-assembly, is not blocked by CARD. Consequently, the preferred dynamics have evolved over time to serve the function.

## 4. Discussion

### 4.1. The Dynamics of ASC Monomer

The loop regions in PYD and CARD and the central linker are the regions with the largest fluctuation. The interdomain bending angle (defined by the Cα atoms of residues 49–69–163) varied from 30° to 160°, and the COM distance distribution between PYD and CARD ranges from 2 to 8 nm, indicating that ASC is relatively flexible. The structure with the greatest COM distance seems to be the inter-conversion state of various compact structures. The representative structures from the cluster analysis of the ASC monomer demonstrate the interdomain flexibility and varied contact interfaces between PYD and CARD.

### 4.2. The Driving Forces of Interdomain Dynamics

The structural features of the linker were characterized to study the origin of ASC interdomain dynamics. It is worth noting that the N-terminus of the linker prefers the helical structure in the ASC monomer, as shown in Figure 4B. Combined with the limited degree of freedom resulting from several prolines in the linker, the linker shows some structural preference. On the other hand, the end-to-end distance of the linker shows that it is flexible both in the ASC linker and in the ASC monomer, as shown in Figure S8A,B. The weak structural preference of the residues in the linker results in absence of a stable secondary structure and thereby promotes the flexibility of the linker. In addition, the TRFDA results show that the central linker can form interactions with both PYD and CARD. The N-terminus of the linker is mainly in contact with PYD, as observed in Figure S3B. The C-terminus of the linker forms interactions primarily with CARD, as observed in Figure S3D, and forms weaker interactions with PYD, as observed in Figure S3B. Furthermore, the correlation map (Figure 3C) also showed that the N-terminus of the linker had motions positively/negatively correlated with the PYD movement and the C-terminus of the linker had motions correlated with the CARD movement. The N-terminus of the linker, physically adjacent to the PYD domain, is more likely to show correlations to the PYD domain than the C-terminus. It was also noted that there are areas of high punctual stress on certain residues between the PYD and CARD domains. This is especially evident in the loop region connecting PYD H2 and H3 and the loop region connecting CARD H5 and H6, as shown in Figure 3A,B. Therefore, weak and transient contacts between linker–linker, linker–domain, and domain–domain seem to be another factor resulting in the interdomain dynamics of the ASC monomer. Although punctual stresses between PYD and CARD were observed, the interactions were relatively dynamic, considering heterogeneous structures generated in the cluster analyses in Figure 2C, which may be neglected compared with the typical interactions responsible for PYD assembly. Therefore, it is not conflicting with the claim on homotypic interactions of death domains proposed in previous experimental reports [24,48].

### 4.3. The Interdomain Dynamics and Its Potential Role in ASC Self-Assembly

Subsequently, we conducted an in-depth study on the potential role of interdomain dynamics in ASC assembly. PCA results show that CARD can rotate relative to PYD. This is important because several adjacent CARDs can rotate their binding interfaces to form homotypic interactions after PYD nucleation and assembly. Furthermore, the torsional angle between PYD and CARD (defined by Cα of residues 81–69–163–177), employed to describe the relative stumbling of PYD and CARD, is widely distributed in the range of −180–180°. The time evolution of the above-mentioned torsional angle shows that the angle sometimes oscillates frequently between −180° and 180°, indicating the interdomain stumbling of the ASC monomer, which is consistent with the PCA results. The rotation may be related to the transient contacts involving the linker and domains. Some interactions were observed in one conformation, while others were observed in other conformations. In other words, these transient interactions were not strong enough to stabilize ASC in one single structure during the entire simulation, yielding structural transitions. In addition, the interdomain rotation appears to be correlated with the dynamic variation of helical probability of residues 90–94 in the linker. Given the flexibility of the linker, CARD can access many regions around PYD. However, the helices involved in the Type I interactions in PYD, namely H1–H4, are inaccessible to CARD. The interdomain spatial restrain is highly related to the properties of the linker, which is flexible but also has structural preferences, such as the turn-type structural preference in the N-terminal region of the linker and geometry limitation of prolines in the linker. Taken together, the structural features of the linker and the weak interdomain interplays lead to the ASC monomer favoring interdomain dynamics that seem to benefit ASC assembly and ultimately the assembly of the inflammasome complex.

## 5. Conclusions

With the MD simulation on the ASC monomer and the isolated linker, we analyzed the origin of the interdomain dynamics of ASC. According to the PCA results, the interdomain dynamics stem from the semi-flexible linker since the ASC monomer showed similar motions as the linker. Additionally, the residues at the N-terminus of the linker show a weak tendency to form a turn-type structure, one possible factor driving the interdomain rotation. ASC is observed to show evident interdomain dynamics, but the dynamics are not entirely free to adopt diverse organizations for the two domains. Analysis of the spatial restraint of CARD suggested that it is likely to populate in certain positions with the Type I interface of PYD being exposed. Therefore, such positions contribute to the subsequent assembly of inflammasome complexes. These findings can be verified by further assembly simulations of more complex systems. With more information on the dynamic behaviors of ASC, we will be able to reveal the activation mechanism of the inflammatory signaling pathway and target ASC to regulate the immune response in inflammasome-related disorders.

## Data Availability

Publicly available datasets were analyzed in this study. This data can be found here: https://tinyurl.com/25jtdhuk (accessed on 28 May 2023).

## Supplementary Materials

The following supporting information can be downloaded at: https://www.mdpi.com/article/10.3390/biology12060796/s1, Figure S1: The distribution of the torsional angle defined by residues 81–69–163–177 and the bending angle defined by residues 49–69–163; Figure S2: Time-resolved force distribution analysis (TRFDA) between domains PYD and CARD; Figure S3: Time-resolved force distribution analysis (TRFDA) between the linker with either PYD or CARD; Figure S4: Network of ASC monomer obtained from correlation data; Figure S5: Relative solvent accessible surface area (SASA) for all helices; Figure S6: The core analysis for the linker of ASC, for both systems: ASC linker and monomer; Figure S7: Contact map and correlation analysis; Figure S8. The linker flexibility; Table S1: The residue ranges of helices applied in Figure S5.

## References

[1] Agostini L., Martinon F., Burns K., McDermott M.F., Hawkins P.N., Tschopp J. (2004). NALP3 Forms an IL-1β-Processing Inflammasome with Increased Activity in Muckle-Wells Autoinflammatory Disorder. Immunity.

[2] Franchi L., Eigenbrod T., Muñoz-Planillo R., Nuñez G. (2009). The Inflammasome: A Caspase-1-Activation Platform That Regulates Immune Responses and Disease Pathogenesis. Nat. Immunol..

[3] Hornung V., Ablasser A., Charrel-Dennis M., Bauernfeind F., Horvath G., Caffrey D.R., Latz E., Fitzgerald K.A. (2009). AIM2 Recognizes Cytosolic DsDNA and Forms a Caspase-1-Activating Inflammasome with ASC. Nature.

[4] Srinivasula S.M., Poyet J.-L., Razmara M., Datta P., Zhang Z., Alnemri E.S. (2002). The PYRIN-CARD Protein ASC Is an Activating Adaptor for Caspase-1. J. Biol. Chem..

[5] Martinon F., Burns K., Tschopp J. (2002). The Inflammasome: A Molecular Platform Triggering Activation of Inflammatory Caspases and Processing of ProIL-β. Mol. Cell.

[6] Martinon F., Hofmann K., Tschopp J. (2001). The Pyrin Domain: A Possible Member of the Death Domain-Fold Family Implicated in Apoptosis and Inflammation. Curr. Biol..

[7] Franchi L., Muñoz-Planillo R., Núñez G. (2012). Sensing and Reacting to Microbes through the Inflammasomes. Nat. Immunol..

[8] Dick M.S., Sborgi L., Rühl S., Hiller S., Broz P. (2016). ASC Filament Formation Serves as a Signal Amplification Mechanism for Inflammasomes. Nat. Commun..

[9] McConnell B.B., Vertino P.M. (2004). TMS1/ASC: The Cancer Connection. Apoptosis.

[10] Drexler S.K., Bonsignore L., Masin M., Tardivel A., Jackstadt R., Hermeking H., Schneider P., Gross O., Tschopp J., Yazdi A.S. (2012). Tissue-Specific Opposing Functions of the Inflammasome Adaptor ASC in the Regulation of Epithelial Skin Carcinogenesis. Proc. Natl. Acad. Sci. USA.

[11] Liu W., Luo Y., Dunn J.H., Norris D.A., Dinarello C.A., Fujita M. (2013). Dual Role of Apoptosis-Associated Speck-Like Protein Containing a CARD (ASC) in Tumorigenesis of Human Melanoma. J. Investig. Dermatol..

[12] Allen I.C., TeKippe E.M., Woodford R.-M.T., Uronis J.M., Holl E.K., Rogers A.B., Herfarth H.H., Jobin C., Ting J.P.-Y. (2010). The NLRP3 Inflammasome Functions as a Negative Regulator of Tumorigenesis during Colitis-Associated Cancer. J. Exp. Med..

[13] Wen H., Gris D., Lei Y., Jha S., Zhang L., Huang M.T.-H., Brickey W.J., Ting J.P.-Y. (2011). Fatty Acid–Induced NLRP3-ASC Inflammasome Activation Interferes with Insulin Signaling. Nat. Immunol..

[14] Martinon F., Pétrilli V., Mayor A., Tardivel A., Tschopp J. (2006). Gout-Associated Uric Acid Crystals Activate the NALP3 Inflammasome. Nature.

[15] Venegas C., Kumar S., Franklin B.S., Dierkes T., Brinkschulte R., Tejera D., Vieira-Saecker A., Schwartz S., Santarelli F., Kummer M.P. (2017). Microglia-Derived ASC Specks Cross-Seed Amyloid-β in Alzheimer’s Disease. Nature.

[16] Broderick L., De Nardo D., Franklin B.S., Hoffman H.M., Latz E. (2015). The Inflammasomes and Autoinflammatory Syndromes. Annu. Rev. Pathol. Mech. Dis..

[17] Strowig T., Henao-Mejia J., Elinav E., Flavell R. (2012). Inflammasomes in Health and Disease. Nature.

[18] Balci-Peynircioglu B., Waite A.L., Schaner P., Taskiran Z.E., Richards N., Orhan D., Gucer S., Ozen S., Gumucio D., Yilmaz E. (2008). Expression of ASC in Renal Tissues of Familial Mediterranean Fever Patients with Amyloidosis: Postulating a Role for ASC in AA Type Amyloid Deposition. Exp. Biol. Med..

[19] Baroja-Mazo A., Martín-Sánchez F., Gomez A.I., Martínez C.M., Amores-Iniesta J., Compan V., Barberà-Cremades M., Yagüe J., Ruiz-Ortiz E., Antón J. (2014). The NLRP3 Inflammasome Is Released as a Particulate Danger Signal That Amplifies the Inflammatory Response. Nat. Immunol..

[20] Kumar M., Roe K., Orillo B., Muruve D.A., Nerurkar V.R., Gale M., Verma S. (2013). Inflammasome Adaptor Protein Apoptosis-Associated Speck-like Protein Containing CARD (ASC) Is Critical for the Immune Response and Survival in West Nile Virus Encephalitis. J. Virol..

[21] De Alba E. (2009). Structure and Interdomain Dynamics of Apoptosis-Associated Speck-like Protein Containing a CARD (ASC). J. Biol. Chem..

[22] Bryan N.B., Dorfleutner A., Kramer S.J., Yun C., Rojanasakul Y., Stehlik C. (2010). Differential Splicing of the Apoptosis-Associated Speck like Protein Containing a Caspase Recruitment Domain (ASC) Regulates Inflammasomes. J. Inflamm..

[23] Sahillioglu A.C., Sumbul F., Ozoren N., Haliloglu T. (2014). Structural and Dynamics Aspects of ASC Speck Assembly. Structure.

[24] Lu A., Magupalli V.G., Ruan J., Yin Q., Atianand M.K., Vos M.R., Schröder G.F., Fitzgerald K.A., Wu H., Egelman E.H. (2014). Unified Polymerization Mechanism for the Assembly of Asc-Dependent Inflammasomes. Cell.

[25] Nambayan R.J.T., Sandin S.I., Quint D.A., Satyadi D.M., de Alba E. (2019). The Inflammasome Adapter ASC Assembles into Filaments with Integral Participation of Its Two Death Domains, PYD and CARD. J. Biol. Chem..

[26] De Alba E. (2019). Structure, Interactions and Self-Assembly of ASC-Dependent Inflammasomes. Arch. Biochem. Biophys..

[27] Marleaux M., Anand K., Latz E., Geyer M. (2020). Crystal Structure of the Human NLRP9 Pyrin Domain Suggests a Distinct Mode of Inflammasome Assembly. FEBS Lett..

[28] Ha H.J., Park H.H. (2020). Crystal Structure of the Human NLRP9 Pyrin Domain Reveals a Bent N-Terminal Loop That May Regulate Inflammasome Assembly. FEBS Lett..

[29] De Alba E. (2020). The Mysterious Role of the NLRP9 Pyrin Domain in Inflammasome Assembly. FEBS Lett..

[30] Sharma M., de Alba E. (2021). Structure, Activation and Regulation of NLRP3 and AIM2 Inflammasomes. Int. J. Mol. Sci..

[31] Berendsen H.J.C., van der Spoel D., van Drunen R. (1995). GROMACS: A Message-Passing Parallel Molecular Dynamics Implementation. Comput. Phys. Commun..

[32] Pall S., Abraham M.J., Kutzner C., Hess B., Lindahl E. (2015). Tackling Exascale Software Challenges in Molecular Dynamics Simulations with GROMACS. Lecture Notes in Computer Science (including Subseries Lecture Notes in Artificial Intelligence and Lecture Notes in Bioinformatics).

[33] Abraham M.J., Murtola T., Schulz R., Páall S., Smith J.C., Hess B., Lindah E. (2015). Gromacs: High Performance Molecular Simulations through Multi-Level Parallelism from Laptops to Supercomputers. SoftwareX.

[34] Darden T., York D., Pedersen L. (1993). Particle Mesh Ewald: An N⋅log(N) Method for Ewald Sums in Large Systems. J. Chem. Phys..

[35] Essmann U., Perera L., Berkowitz M.L., Darden T., Lee H., Pedersen L.G. (1995). A Smooth Particle Mesh Ewald Method. J. Chem. Phys..

[36] Jo S., Kim T., Iyer V.G., Im W. (2008). CHARMM-GUI: A Web-Based Graphical User Interface for CHARMM. J. Comput. Chem..

[37] Lee J., Cheng X., Swails J.M., Yeom M.S., Eastman P.K., Lemkul J.A., Wei S., Buckner J., Jeong J.C., Qi Y. (2016). CHARMM-GUI Input Generator for NAMD, GROMACS, AMBER, OpenMM, and CHARMM/OpenMM Simulations Using the CHARMM36 Additive Force Field. J. Chem. Theory Comput..

[38] Hess B., Bekker H., Berendsen H.J.C., Fraaije J.G.E.M. (1997). LINCS: A Linear Constraint Solver for Molecular Simulations. J. Comput. Chem..

[39] Murtagh F., Legendre P. (2014). Ward’s Hierarchical Agglomerative Clustering Method: Which Algorithms Implement Ward’s Criterion?. J. Classif..

[40] Grant B.J., Rodrigues A.P.C., ElSawy K.M., McCammon J.A., Caves L.S.D. (2006). Bio3d: An R Package for the Comparative Analysis of Protein Structures. Bioinformatics.

[41] Costescu B.I., Gräter F. (2013). Time-Resolved Force Distribution Analysis. BMC Biophys..

[42] Zhou J., Aponte-Santamaría C., Sturm S., Bullerjahn J.T., Bronowska A., Gräter F. (2015). Mechanism of Focal Adhesion Kinase Mechanosensing. PLoS Comput. Biol..

[43] Lindahl E., Hess B., van der Spoel D. (2001). GROMACS 3.0: A Package for Molecular Simulation and Trajectory Analysis. Mol. Model. Annu..

[44] McGibbon R.T., Beauchamp K.A., Harrigan M.P., Klein C., Swails J.M., Hernández C.X., Schwantes C.R., Wang L.-P., Lane T.J., Pande V.S. (2015). MDTraj: A Modern Open Library for the Analysis of Molecular Dynamics Trajectories. Biophys. J..

[45] Touw W.G., Baakman C., Black J., Te Beek T.A.H., Krieger E., Joosten R.P., Vriend G. (2015). A Series of PDB-Related Databanks for Everyday Needs. Nucleic Acids Res..

[46] Kabsch W., Sander C. (1983). Dictionary of Protein Secondary Structure: Pattern Recognition of Hydrogen-Bonded and Geometrical Features. Biopolymers.

[47] Oroz J., Barrera-Vilarmau S., Alfonso C., Rivas G., De Alba E. (2016). ASC Pyrin Domain Self-Associates and Binds NLRP3 Protein Using Equivalent Binding Interfaces*. J. Biol. Chem..

[48] Weber C.H., Vincenz C. (2001). The Death Domain Superfamily: A Tale of Two Interfaces?. Trends Biochem. Sci..

