# Supplementary material for: Key Factors Regulating the Interdomain Dynamics May Contribute to the Assembly of ASC
Source: Biology (Basel). 2023 May 31;12(6):796. doi: 10.3390/biology12060796 (PMC10294833; doi:10.3390/biology12060796)
Supplement: Supplementary file 1 [file biology-12-00796-s001.zip › biology-2406196-supplementary.pdf]

Supplementary Materials

for

Key Factors Regulating the Interdomain Dynamics May Contribute to the Assembly of ASC

Tongtong Li [1], Laura I. Gil Pineda [1,2], Amy O. Stevens [1] and Yi He [1,*]

[1] Department of Chemistry & Chemical Biology, The University of New Mexico, Albuquerque, NM 87131, USA
[2] Department of Biochemistry, Virginia Tech, 340 West Campus Dr, Blacksburg, VA 24061, USA
* Correspondence: yihe@unm.edu

ASC Monomer is Highly Dynamic Two angles were used to explore the interdomain dynamics of the ASC monomer. As observed in Figure S1A, the torsional angle defined with residues of 81–69-–163–177 can rotate in the range of –180°–180°. This implies that the domains are flexible enough to adopt various structures. The bending angle defined with residues 49–69–163 spans from 50° to 150°, suggesting that these two domains can open and close relatively freely. But the fully open (bending angle of 180°) or closed state (bending angle of 0°) is inaccessible, so the probability of a compact or extended state is very low. Time-resolved force distribution analysis (TRFDA) was conducted to assess the transient interactions in ASC interdomain dynamics. TRFDA between PYD–CARD (Figure S2), PYD–linker (Figure S3A,B), and CARD–linker (Figure S3C,D), was carried out to identify residues enduring high punctual stresses. The complete TRFDA between PYD and CARD is shown in Figure S2, including the major fragments with high punctual stresses in Figure 3A,B. It is interesting to note that the linker can form interactions with either PYD or CARD (Figure S3). In particular, residues 101–109 (Figure S3D) formed interactions with residues 113–119 and residues 160–167 in CARD (Figure S3C) before 0.5 µs. During the last 0.4 µs, residues 101–109 (Figure S3B) formed interactions with residues 51–55 in PYD (Figure S3A). For example, Lys109 may form an interaction with either Asp54 in PYD or Asp116 in CARD.

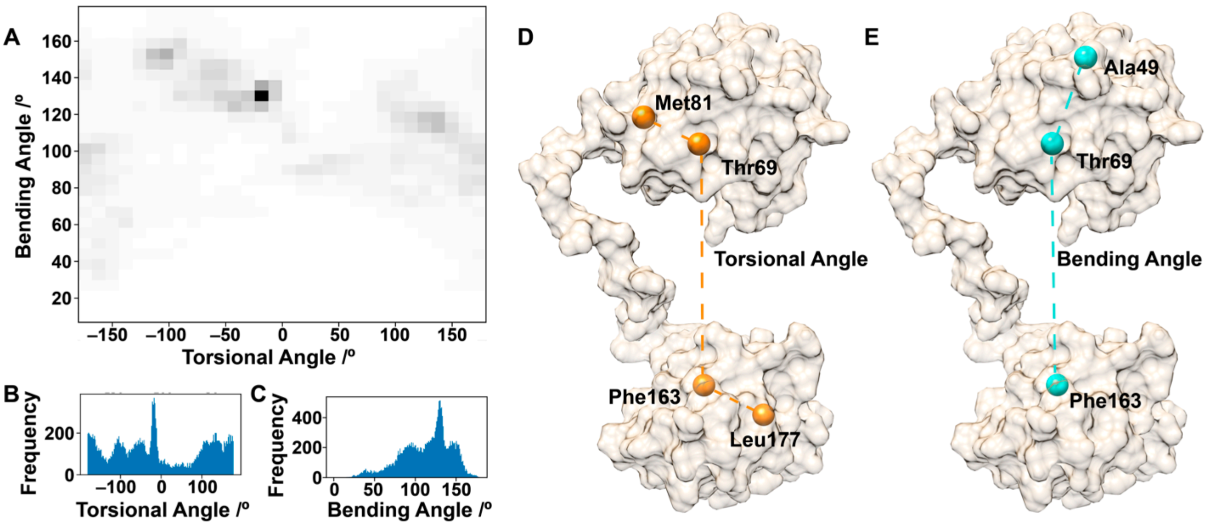

Figure S1. The torsional angle distribution (panel **(B)** and x-axis in panel **(A)**) and the bending angle distribution (panel **(C)** and y-axis in panel **(A)**). The torsional angle is defined by Cα atoms of residues 81–69–163–177 displayed in panel **(D)**. The bending angle is defined by Cα atoms of residues 49–69–163 labeled in panel **(E)**.

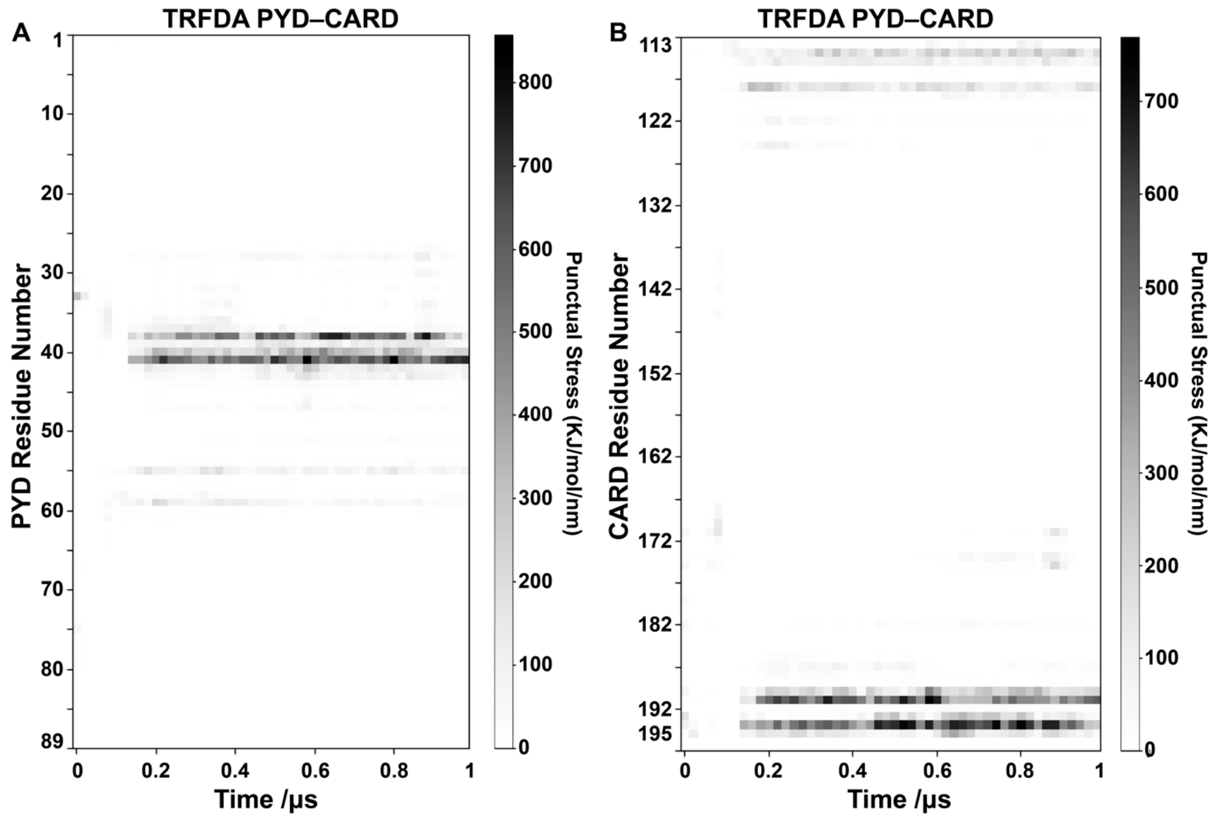

Figure S2. Time-resolved force distribution analysis (TRFDA) between domains PYD and CARD. **(A)** The punctual stress on PYD residues. **(B)** The punctual stress on CARD residues.

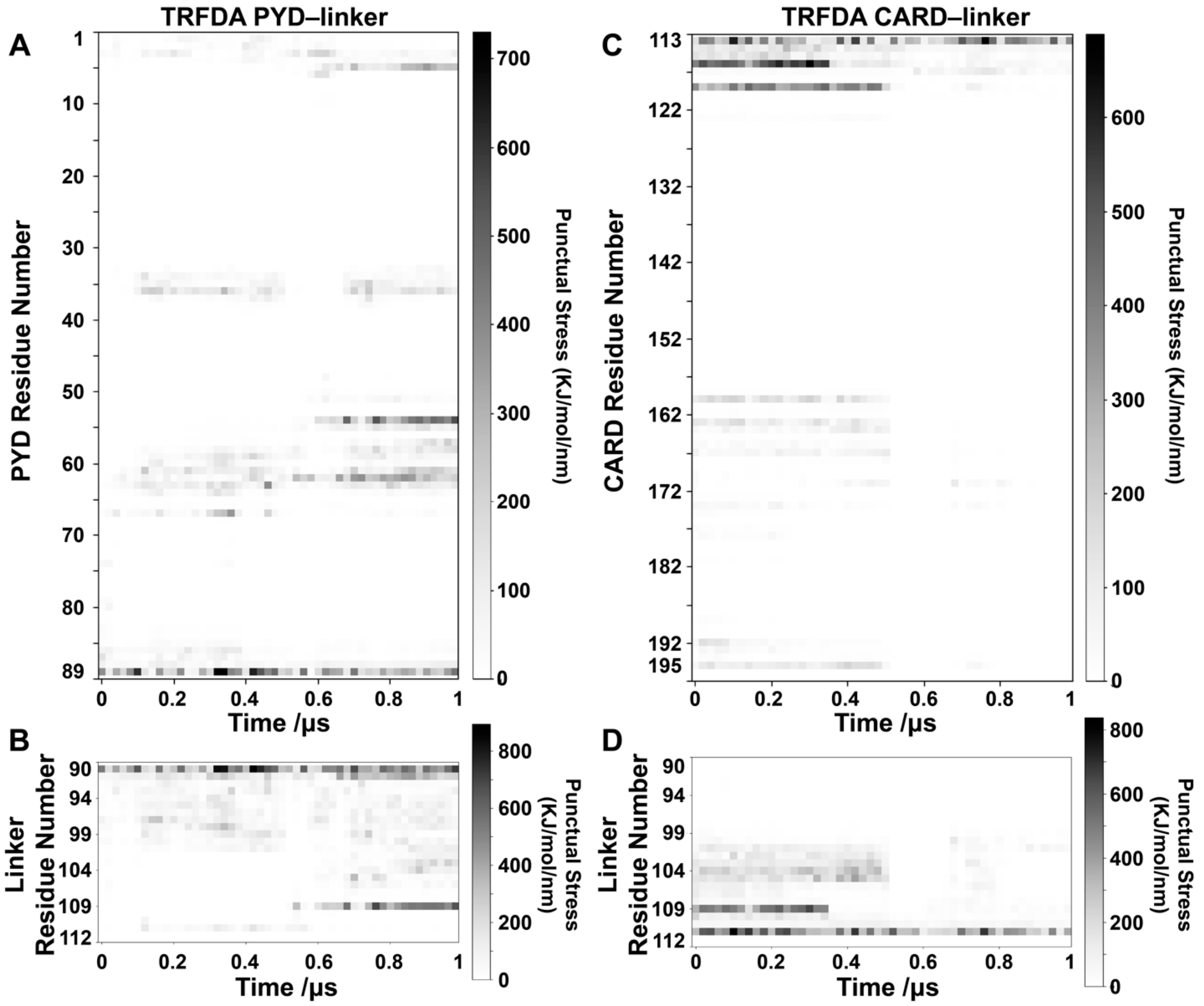

Figure S3. Time-resolved force distribution analysis (TRFDA) between the linker with either PYD or CARD. TRFDA between the linker and PYD, with punctual stress on PYD residues **(A)** and the linker residues **(B)**. TRFDA between the linker and CARD, with punctual stress on CARD residues **(C)** and the linker residues **(D)**.

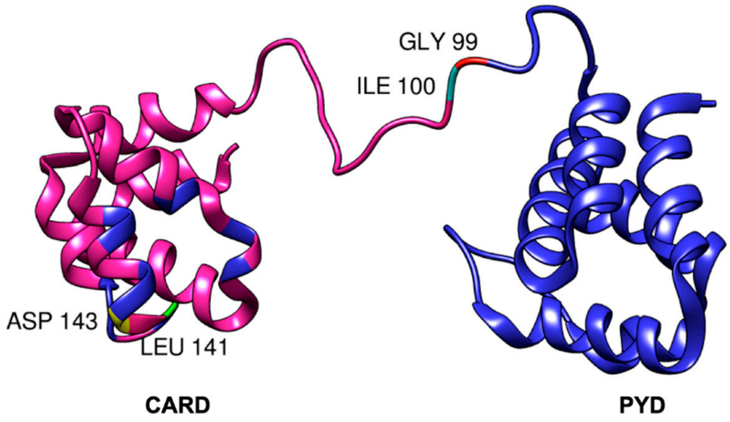

Figure S4. Network of ASC monomer obtained from correlation data. Notice PYD residues are all correlated (blue), in contrast to CARD. Additionally, some residues are not correlated to either domain: 99-red, 100-cyan, 141-green, and 143-yellow.

The relative solvent-accessible surface area (SASA) for all helices of PYD and CARD (Figure S5) was calculated to further study the exposure of each helix in detail. Residues with a helix propensity cutoff of 0.35 are considered part of a helix, as shown in Table S1. The SASA value for each helix can then be used to calculate the fraction relative to the maximum SASA (indicating little contact between PYD and CARD and the buried surface is mostly from contact within the internal domain), which was averaged over all frames. In general, the SASA of a helix is determined by the helical packing in each domain. If a helix in one ASC domain also contacts the linker or the other domain, SASA will decrease. It is true for the relative SASA values shown in Figure S5. Most interdomain interactions are located in the loops, especially the PYD H2–H3 loop, rather than helices. This results in little change in the SASA of most helices, approximately a 10% reduction. But this is not the case for H3 in PYD, H5 and H6 in CARD. In PYD, the SASA of H3 is variable, which may be related to dynamic interactions involving the H2–H3 loop. In CARD, H5 and H6 interact with the linker and the PYD H2–H3 loop according to the contact map, which explains the variation in SASA and may lead to relative stumbling between domains.

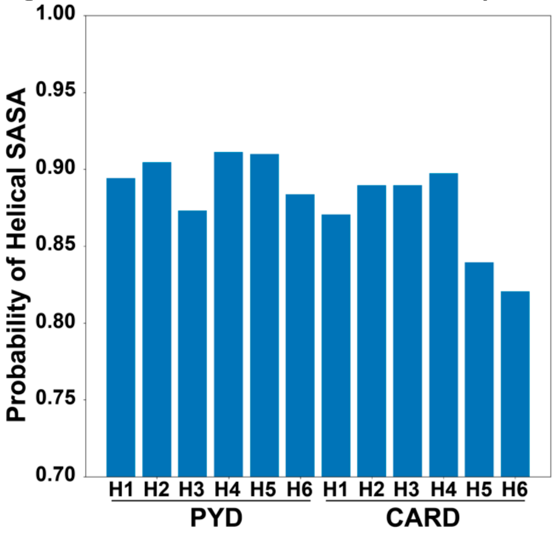

Figure S5. Relative solvent accessible surface area (SASA) for all helices. Probability 1 represents conformers with little packing between the helix and the other domain or the linker.

Table S1. The residue ranges of helices applied in Figure. S5.

PYD Helices	Residue range		CARD Helices	Residue range	
H1	4	14	H1	113	125
H2	17	29	H2	129	136
H3	41	46	H3	143	151
H4	49	60	H4	155	168
H5	62	76	H5	171	184
H6	79	89	H6	186	192

Linker Has Structural Preferences but Is Still Highly Dynamic

In Figure 4B, the N-terminus of the linker shows the helical preference to some extent, however, the ten-residue core displayed in Figure S6 actually covers residues Gly94–Pro103. A contact map analysis (Figure S7A) and a correlation analysis (Figure S7B) were performed. As shown in Figure S7B, a negative correlation between the first 8 residues and the last 7 residues could be identified. In Figure S8, the end-to-end distance (between residues 90 and 112) and the degree of opening/closing of the linker (dihedral angle defined by residues 90–99–100–112) were investigated in both the linker and the ASC monomer. The end-to-end distance is widely distributed, but the population of either very long or very short distances and the frequency of the distance variation decreased in the monomer. The dihedral angle is also widely distributed, but the probability of being close to zero is very low, with zero indicating that PYD and CARD are in contact in the conformer. The wide distribution of the distance and the dihedral angle suggests that the linker is flexible, and the difference between the ASC linker and the linker in the ASC monomer is obviously due to steric clashes between PYD and CARD.

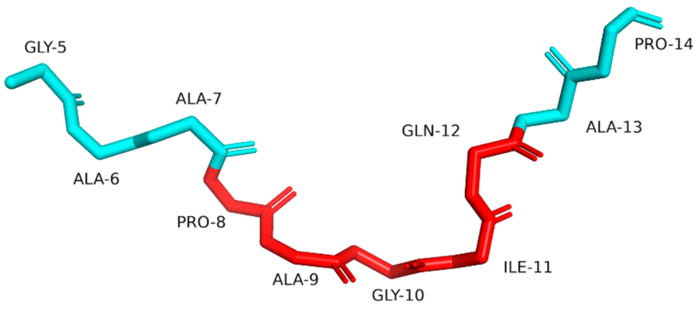

Figure S6. The core analysis for the linker of ASC, for both systems: ASC linker and monomer. In red is the five-residue core that is part of the ten-residue core in cyan. Gly5 and Pro14 in the linker are Gly94 and Pro103 in the ASC monomer, respectively.

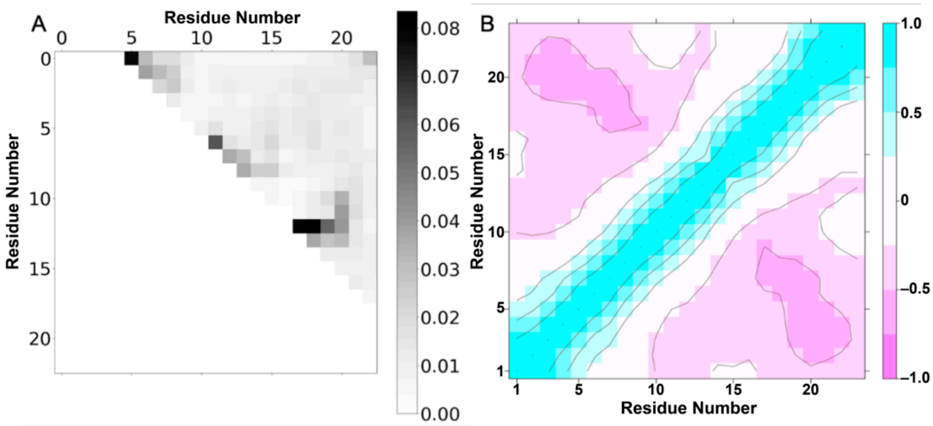

Figure S7. (A) Contact map corresponding to all 27 µs of ASC linker simulation. Contacts present for most of the simulation (higher probability) will be shown in a darker color (black). **(B)** Correlation analysis corresponding to all 27 µs of ASC linker simulations. Light blue and pink indicate positive and negative correlations, respectively.

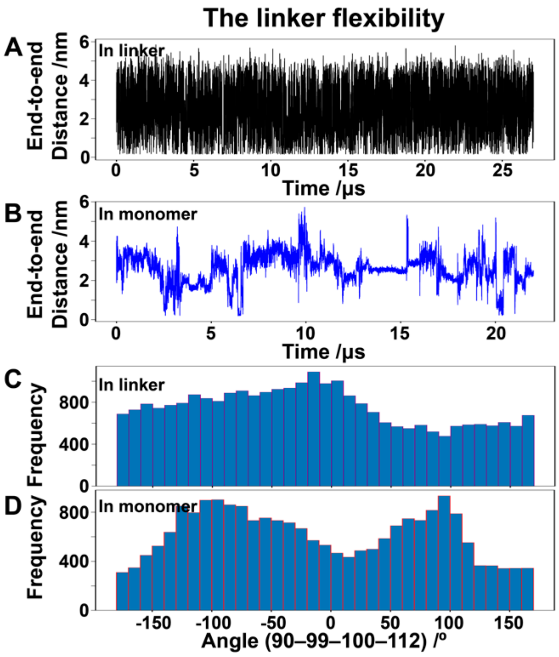

Figure S8. The linker flexibility. The end-to-end distance describes the distance between residues 90 and 112 in the isolated linker **(A)** and in the ASC monomer **(B)**. The angle distribution describes the degree of freedom of the dihedral angle defined by residues 90, 99, 100, and 112 in the isolated linker **(C)** and in the ASC monomer **(D)**.
